# Physical Activity Levels of a Multi-Ethnic Population of Middle-Aged Men Living in Saudi Arabia and Factors Associated With Physical Inactivity

**DOI:** 10.3389/ijph.2021.1604328

**Published:** 2022-02-07

**Authors:** Nora A. AlFaris, Naseem M. Alshwaiyat, Jozaa Z. AlTamimi, Reham I. Alagal, Hamid A. Al-Jamal, Nora M. AlKehayez

**Affiliations:** ^1^ Department of Physical Sports Sciences, College of Education, Princess Nourah bint Abdulrahman University, Riyadh, Saudi Arabia; ^2^ School of Nutrition and Dietetics, Faculty of Health Sciences, Universiti Sultan Zainal Abidin, Terengganu, Malaysia; ^3^ School of Biomedicine, Faculty of Health Sciences, Universiti Sultan Zainal Abidin, Terengganu, Malaysia

**Keywords:** body mass index, physical activity, physical inactivity, middle-aged men, multi-ethnic, Saudi Arabia

## Abstract

**Objectives:** This study investigated physical activity levels and factors related to physical inactivity in a multi-ethnic population of middle-aged men living in Saudi Arabia.

**Methods:** This is a cross-sectional study in which 1,800 men aged 36–59 years old from Riyadh, Saudi Arabia participated. The Global Physical Activity Questionnaire was used to assess the physical activity levels. The weight and height were measured and used to calculate the body mass index.

**Results:** Among all participants, 35.3% are physically inactive. The participants with the lowest and highest rates of physical inactivity were from the Philippines (15.5%) and Saudi Arabia (57.8%), respectively. This study suggests that the risk of physical inactivity among participants is positively or negatively affected by various factors, including nationality, residency period in Saudi Arabia, living with/without family, education level, monthly income, and body mass index.

**Conclusion:** The prevalence of physical inactivity is relatively high among middle-aged men in Saudi Arabia. The findings revealed significant differences in physical activity levels based on nationality, other sociodemographic variables and body weight status.

## Introduction

Physical activity is any voluntary movement of body skeletal muscles that requires more energy expenditure than the resting metabolic rate [1]. Doing physical activity on a regular basis has long been acknowledged as a protective factor against many common chronic diseases, including coronary heart disease, obesity, and type 2 diabetes [2]. Physical activity also aids in the improvement of mental health and overall quality of life [3, 4]. In contrast, physical inactivity is known as the absence of moderate to high physical activity in a person’s lifestyle [5]. Current evidence emphasized that over one-fourth of the world’s adults are physically inactive [6]. Physical inactivity has been identified as a global public health issue connected to increased morbidity and mortality among adults [7, 8]. Accordingly, the World Health Organization (WHO) has set a goal of reducing the global incidence of physical inactivity in adults and adolescents by 15% by 2030 [5].

Saudi Arabia has passed through a socioeconomic transformation in the last few decades that coincide with changes in the lifestyles of the community toward sedentary behaviors as a result of urbanization and motorization [9]. These modifications in the lifestyle are occurring parallel with the rising prevalence of obesity and other chronic diseases among the Saudi population [10]. The upsurge in the prevalence of obesity and chronic diseases in Saudi Arabia is owing to the increased consumption of unhealthy foods and decreased physical activity [11–13].

Physical inactivity rates among adults vary dramatically between countries and among subpopulations. Physical inactivity among adults is highest in the Eastern Mediterranean, the Americas, Europe, and the Western Pacific region, while it is lowest in South-East Asia [14]. Numerous factors, including economic development, transportation forms, technology usage, and cultural values, could influence these rates [15]. Saudi Arabia has seen a massive arrival of migrant workers in recent decades, most of whom were young and middle-aged men from many Middle Eastern and Asian countries [16]. Non-Saudi residents formed about 31% of the population living in Saudi Arabia; 70% of them were males [17]. Physical activity levels among men from various countries living in Saudi Arabia varies significantly [18]. These disparities could be due to diversity in lifestyle aspects, including work type, transportation, leisure time activities [14]. Fortunately, investigating these disparities in physical activity levels can aid in identifying and implementing effective strategies for promoting physical activity in various population subgroups. This study was conducted to assess physical activity levels and factors associated with physical inactivity among a multi-ethnic sample of middle-aged men living in Saudi Arabia.

## Methods

### Study Design and Participants

This study is part of a research project named the Relationship between Obesity, physical Activity, and Dietary pattern among men in Saudi Arabia (ROAD-KSA) Project. It is a cross-sectional study designed to evaluate the prevalence of obesity, physical activity levels, and dietary patterns among young and middle-aged men living in Saudi Arabia. This study was conducted in Riyadh, Saudi Arabia.

Using a stratified clustered sampling technique based on geographic locations, the participants in this study were recruited at random from public sites in Riyadh. The inclusion criteria for participation includes men aged 36–59 years, living in Riyadh, being free of any physical impairment, and having a single nationality of Saudi Arabia, Egypt, Yemen, Syria, Jordan, Sudan, Turkey, Pakistan, Afghanistan, India, Bangladesh, or the Philippines. Before taking part in this study, participants signed an informed consent according to Helsinki Declaration. Ethical clearance for this study was approved by the research ethics committee of Princess Nourah bint Abdulrahman University in Riyadh, Saudi Arabia.

### Sociodemographic Characteristics

Sociodemographic data were collected using face-to-face interviews. The collected sociodemographic information includes nationality, age, residency period in Saudi Arabia, household type, marital status, educational level, and monthly income.

### Weight and Height Measurement

A calibrated digital weight scale was used to measure the weight to the nearest 0.1 kg while wearing light clothing and no shoes. Besides, a calibrated portable stadiometer was used to measure the height to the nearest 0.1 cm in full standing posture without shoes. Body mass index (BMI) was computed by dividing weight (kg) by height square (m^2^) [19].

### Physical Activity Measurement

The Global Physical Activity Questionnaire (GPAQ) version 2.0 was used to measure physical activity in three domains: work, transportation and recreation, respectively [20]. The GPAQ has acceptable reliability and validity for measuring adult physical activity [21]. The GPAQ was developed by WHO for physical activity surveillance in large population surveys and comprised of 16 questions: six questions assessed work-related physical activities, three questions assessed transportation-related physical activities, and six questions assessed recreation-related physical activities. The GPAQ consists of an additional question about typical daily times spent on sedentary behaviors [22]. Sedentary behaviors are defined as sitting or reclining at work or home, including time spent travelling by vehicles, reading or watching television, but do not include time spent sleeping [22]. The intensity of physical activities explored using GPAQ is categorized into moderate and vigorous-intensity physical activities. Vigorous-intensity activities are defined as activities that require hard physical effort and cause large increases in breathing or heart rate. Moderate-intensity activities are defined as activities that require moderate physical effort and cause small increases in breathing or heart rate [22]. Metabolic Equivalent of Tasks (METs) is a standard unit of measurement for expressing the intensity of physical activity. When calculating a person’s overall energy expenditure using GPAQ data, 4 METs are given to the time spent in moderate-intensity physical activities, and 8 METs are given to the time spent in vigorous-intensity physical activities [22]. The first and third domains of the GPAQ asked about the number of typical weekly days and typical daily times spent on vigorous and moderate-intensity activities related to work and recreation, respectively. The second domain of the GPAQ asked about the number of typical weekly days and typical daily times spent on moderate-intensity activities related to transportation [22].

A pilot study was carried out to assess the reliability and validity of the GPAQ. Pilot study data were collected from 60 participants recruited from the target population and were not included in the study sample. To determine test-retest reliability, the GPAQ was administered twice within 2 weeks apart using face-to-face interviews. The total moderate to vigorous physical activity (MVPA) METs-minutes value is calculated from the sum of all METs-minutes per week from moderate and vigorous-intensity physical activities performed at work, transport, and recreation [22]. Then, the average of daily MVPA minutes is calculated. To examine validity, participants wore accelerometers (ActiGraph GT3X, Pensacola, United States) for seven consecutive days on the right hip except during sleep and water-based activities. ActiLife version 6.13.3 (2016) software was used to obtain data from the accelerometer. Freedson and colleagues cut-off points were used to classify time spent in sedentary [<100 counts per minute (CPM)], light (100–1951 CPM), moderate (1952–5724 CPM), and vigorous (>5724 CPM) physical activities for adults [23]. The average daily MVPA minutes is calculated. Test-retest data were collected from all participants recruited in the pilot study, while valid accelerometer data (≥10 h/day of wear-time for at least 4 days) were collected from 54 participants and compared with the first administered GPAQ. A non-wear period was defined as 60 consecutive minutes of zero counts. The Spearman’s correlation between the GPAQ test and retest for MVPA was strong (r = 0.92, *p* < 0.05). The Spearman’s correlation between the GPAQ and accelerometry MVPA was moderate (r = 0.64, *p* < 0.05).

Face-to-face interviews conducted by professional researchers were used to obtain data on physical activity. According to the GPAQ analysis guide, The GPAQ categorized physical activity into three levels (high, moderate and low) based on specific criteria [22]. The physical activity level is classified as high if a person reported vigorous-intensity activity on at least 3 days, with a minimum of 1500 MET-minutes per week or seven or more days of any combination of walking or moderate or vigorous-intensity activities, with a minimum of 3000 MET-minutes per week. The level of physical activity is classified as moderate if a person reported 3 or more days of vigorous-intensity activity of at least 20 min per day or 5 or more days of moderate-intensity activity of at least 30 min per day or 5 or more days of any combination of walking, moderate- or vigorous-intensity activities achieving a minimum of 600 MET-minutes per week. Otherwise, the level of physical activity is classified as low if the above criteria were not satisfied [22]. The low level of physical activity is considered physical inactivity, whereas the moderate and high levels of physical activity are considered physical activity [5, 24]. Furthermore, the GPAQ data analysis results examined if WHO recommendations for physical activity had been met or not by each participant. WHO recommendations on physical activity for health include doing at least 150 min per week of moderate-intensity physical activity or 75 min per week of vigorous-intensity physical activity or equivalent combination of moderate- and vigorous-intensity physical activity achieving at least 600 MET-minutes per week [22]. Other results generated from the GPAQ data analysis include total daily minutes spent on physical activity, daily minutes spent on various physical activity domains (work, transportation and recreation), daily minutes spent on vigorous and moderate-intensity physical activities, the proportion of daily minutes spent on various physical activity domains from total daily minutes spent on physical activity, the proportion of daily minutes spent on vigorous and moderate-intensity physical activities from total daily minutes spent on physical activity, percent of participants doing no physical activities related to various physical activity domains, and percent of participants doing no physical activities related to vigorous and moderate-intensity physical activities [22].

### Statistical Analysis

For data analysis, IBM SPSS Statistics for Windows (version 26. Armonk, New York, United States, 2019) was used. After stratifying the participants depending on their country, statistical analysis for physical activity levels was carried out for all study sample subgroups. Categorical variables were analyzed by using the Chi-squared test and presented as numbers and percentages. Continuous variables were analyzed by using a one-way ANOVA test and presented as means and standard deviations. Univariate and multivariate logistic regression analyses were performed to detect the factors related to physical inactivity risk. All reported *p* values were made on the basis of two-tailed tests. Differences were considered statistically significant when *p* values <0.05.

## Results

Table 1 shows the sociodemographic characteristics and BMI of the participants. This study comprised 1,800 middle-aged men living in Riyadh, Saudi Arabia, from twelve Middle Eastern and Asian countries. The average age of the participants was 40.9 ± 3.8 years, and they had lived in Saudi Arabia for 13.2 ± 10.6 years on average. The majority of the participants (63.1%) live in non-family households. The sample consisted of 88.1% married men, and the rest of participants were single. About two-thirds of the participants (65.7%) had completed high school or less, while more than half of the participants (55.4%) had a low monthly income (less than 1,000 USD). Finally, participants had a mean BMI of 26.6 ± 3.6.

**TABLE 1:** Sociodemographic characteristics and body mass index of all participants (n = 1,800), Relationship between Obesity, Physical Activity, and Dietary Pattern among Men in Saudi Arabia Project, Saudi Arabia, 2019.

Variables	N/mean	SD/%
Participants Nationality		
Saudi	161	8.9%
Egyptian	161	8.9%
Yemeni	115	6.4%
Syrian	157	8.7%
Jordanian	170	9.4%
Sudanese	174	9.7%
Turkish	247	13.7%
Pakistani	144	8.0%
Afghan	147	8.2%
Indian	153	8.5%
Bangladeshi	100	5.6%
Filipino	71	3.9%
Age (years)	40.9	3.8
Residency Period in Saudi Arabia (years)	13.2	10.6
Household Type		
Non-family household	1,136	63.1%
Family household	646	36.9%
Marital Status		
Single	214	11.9%
Married	1,586	88.1%
Education Level		
High school or less	1,182	65.7%
College degree or more	618	34.3%
Monthly Income		
Low (<1000 USD)	998	55.4%
High (≥1000 USD)	802	44.6%
Body mass index (kg/m^2^)	26.6	3.6

**FIGURE 1:**
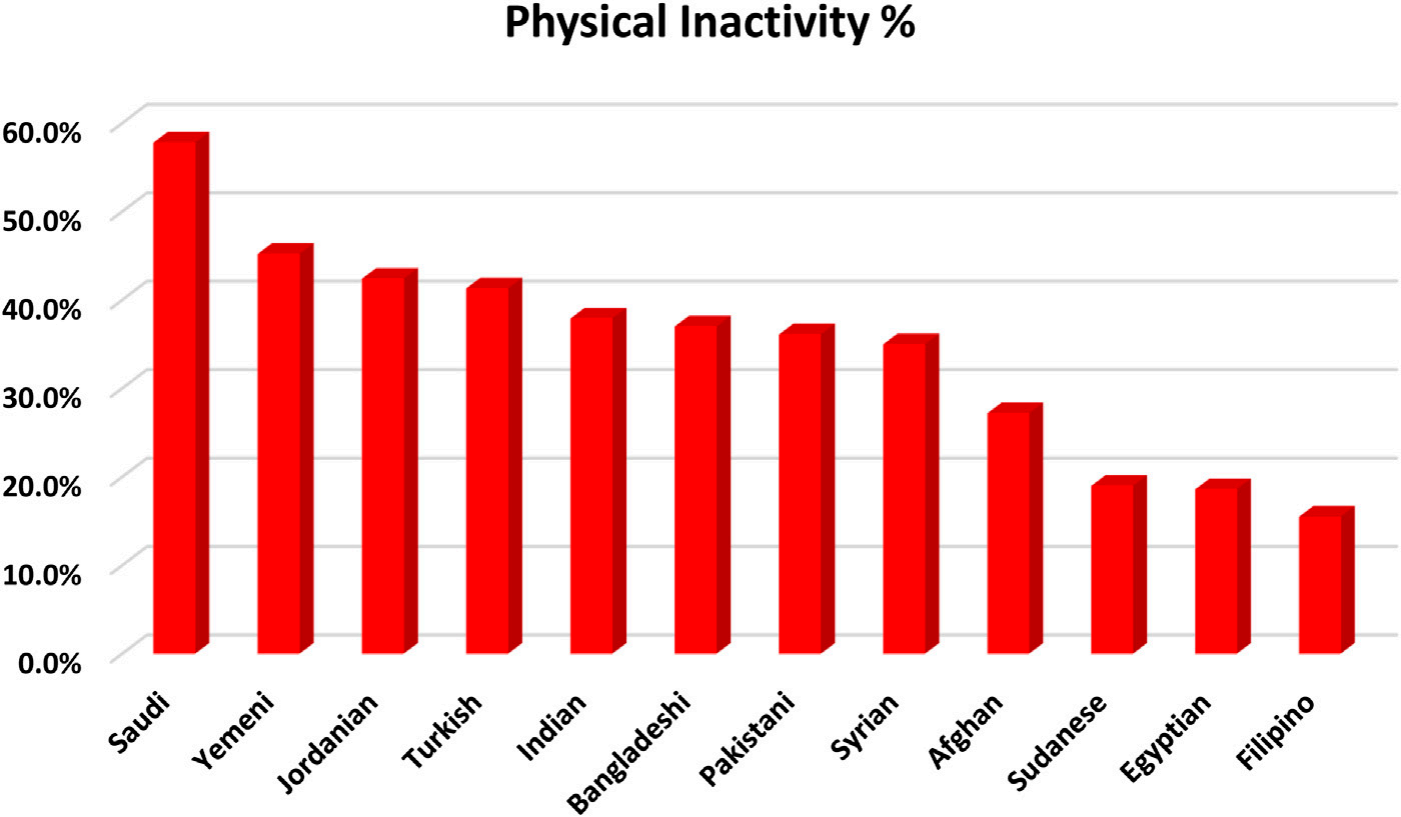
Bar chart illustrating physical inactivity prevalence among the participants stratified based on their nationality, Relationship between Obesity, Physical Activity, and Dietary Pattern among Men in Saudi Arabia Project, Saudi Arabia, 2019.

Table 2 shows the physical activity characteristics of all participants and participants stratified by nationalities. Low physical activity level (physical inactivity) was reported among 35.3% of the participants. Moreover, 25.3% of participants were found with a moderate level of physical activity, while 39.4% were found with a high level of physical activity. Participants with different nationalities differ in the prevalence of physical inactivity (see Figure 1). While the prevalence of physical inactivity among participants from the Philippines was 15.5%, it was 57.8% among participants from Saudi Arabia. About three-quarters of the participants (74.1%) met WHO recommendations for physical activity for health. Participants spent an average of 143.9 ± 186.3 min per day on physical activity. Moreover, the mean daily minutes spent by the participants on work, transport, and recreation-related physical activities were 110.9 ± 167.2 min, 21.4 ± 49.3 min, and 11.6 ± 27.8 min, respectively. Likewise, participants spent an average of 68.5 ± 146.9 min per day on vigorous-intensity active activities and 75.3 ± 108.3 min per day on moderate-intensity physical activities. Finally, participants spent an average of 274.8 ± 186.0 min per day on sedentary behaviors.

**TABLE 2:** Physical activity characteristics of all participants (*n* = 1800) and participants stratified by nationalities, Relationship between Obesity, Physical Activity, and Dietary Pattern among Men in Saudi Arabia Project, Saudi Arabia, 2019.

Variables^a^	Total *n* = 1800	Saudi Arabia *n* = 161	Yemen *n* = 115	Jordan *n* = 170	Turkey *n* = 247	India *n* = 153	Bangladesh *n* = 100
Physical activity levels							
Low	635 (35.3%)	93 (57.8%)	52 (45.2%)	72 (42.4%)	102 (41.3%)	58 (37.9%)	37 (37.0%)
Moderate	456 (25.3%)	38 (23.6%)	25 (21.7%)	71 (41.8%)	86 (34.8%)	40 (26.1%)	27 (27.0%)
High	709 (39.4%)	30 (18.6%)	38 (33.0%)	27 (15.9%)	59 (23.9%)	55 (35.9%)	36 (36.0%)
Participants meeting WHO recommendations^b^	1,333 (74.1%)	85 (52.8%)	74 (64.3%)	120 (70.6%)	178 (72.1%)	103 (67.3%)	81 (81.0%)
Daily minutes spent on activities related to							
Work	110.9 (167.2)	25.1 (65.6)	80.3 (135.2)	16.8 (41.1)	64.2 (94.6)	67.7 (92.2)	109.3 (164.8)
Transport	21.4 (49.3)	10.1 (18.0)	10.9 (33.6)	18.7 (27.1)	15.0 (31.3)	28.9 (72.0)	85.6 (133.1)
Recreation	11.6 (27.8)	16.9 (33.0)	17.5 (59.5)	12.6 (19.6)	6.2 (20.7)	6.4 (18.1)	6.4 (11.7)
Total	143.9 (186.3)	52.1 (87.3)	110.6 (152.8)	48.1 (51.4)	85.4 (103.6)	103.1 (120.1)	201.4 (264.6)
Vigorous activities	68.5 (146.9)	10.2 (31.9)	43.0 (104.4)	9.3 (29.3)	16.4 (51.3)	26.8 (73.7)	74.2 (148.5)
Moderate activities	75.3 (108.3)	41.9 (68.3)	67.6 (104.1)	38.8 (44.2)	69.0 (91.6)	76.3 (108.1)	127.0 (149.8)
Sedentary behaviors	274.8 (186.0)	444.1 (201.1)	631.1 (186.4)	301.8 (142.3)	137.4 (82.1)	157.9 (84.8)	163.8 (100.3)
Proportion of minutes spent in physical activities related to							
Work	0.53 (0.42)	0.30 (0.38)	0.47 (0.45)	0.22 (0.34)	0.57 (0.38)	0.48 (0.41)	0.38 (0.39)
Transport	0.29 (0.36)	0.35 (0.40)	0.28 (0.40)	0.49 (0.40)	0.32 (0.35)	0.44 (0.39)	0.47 (0.40)
Recreation	0.19 (0.31)	0.35 (0.38)	0.25 (0.39)	0.29 (0.35)	0.11 (0.24)	0.08 (0.17)	0.15 (0.31)
Vigorous activities	0.27 (0.38)	0.15 (0.26)	0.28 (0.38)	0.14 (0.29)	0.15 (0.29)	0.15 (0.33)	0.19 (0.32)
Moderate activities	0.73 (0.38)	0.86 (0.26)	0.73 (0.38)	0.86 (0.29)	0.86 (0.29)	0.85 (0.33)	0.81 (0.32)
Percentage of participants doing no physical activities related to							
Work	718 (39.9%)	105 (65.2%)	57 (49.6%)	113 (66.5%)	85 (34.4%)	70 (45.8%)	47 (47.0%)
Transport	758 (42.1%)	93 (57.8%)	68 (59.1%)	52 (30.6%)	110 (44.5%)	46 (30.1%)	20 (20.0%)
Recreation	1,048 (58.2%)	86 (53.4%)	73 (63.5%)	76 (44.7%)	177 (71.7%)	104 (68.0%)	69 (69.0%)
Vigorous activities	1,129 (62.7%)	122 (75.8%)	71 (61.7%)	124 (72.9%)	184 (74.5%)	123 (80.4%)	71 (71.0%)
Moderate activities	305 (16.9%)	44 (27.3%)	26 (22.6%)	16 (9.4%)	48 (19.4%)	37 (24.2%)	7 (7.0%)

Variables^a^	Pakistan n = 144	Syria n = 157	Afghanistan n = 147	Sudan n = 174	Egypt n = 161	Philippines n = 71	*P* value
Physical activity levels							
Low	52 (36.1%)	55 (35.0%)	40 (27.2%)	33 (19.0%)	30 (18.6%)	11 (15.5%)	0.001
Moderate	19 (13.2%)	36 (22.9%)	11 (7.5%)	13 (7.5%)	57 (35.4%)	33 (46.5%)	
High	73 (50.7%)	66 (42.0%)	96 (65.3%)	128 (73.6%)	74 (46.0%)	27 (38.0%)	
Participants meeting WHO recommendations^b^	112 (77.8%)	116 (73.9%)	118 (80.3%)	147 (84.5%)	134 (83.2%)	65 (91.5%)	0.001
Daily minutes spent on activities related to							
Work	210.9 (225.0)	92.3 (146.1)	255.6 (237.6)	207.0 (169.6)	155.5 (202.7)	41.5 (57.0)	0.001
Transport	21.5 (37.0)	12.7 (32.0)	28.9 (39.3)	12.1 (8.9)	14.4 (29.9)	24.3 (25.7)	0.001
Recreation	20.9 (27.6)	20.9 (42.6)	11.2 (16.6)	3.5 (11.4)	8.4 (16.8)	12.9 (14.2)	0.001
Total	253.3 (258.8)	125.8 (156.1)	295.7 (256.7)	222.5 (170.6)	178.2 (206.9)	78.6 (58.1)	0.001
Vigorous activities	169.5 (239.7)	33.3 (79.4)	215.8 (235.7)	125.5 (151.5)	96.0 (165.0)	13.9 (26.8)	0.001
Moderate activities	83.8 (85.3)	92.5 (122.5)	79.8 (96.1)	97.1 (162.2)	82.3 (121.2)	64.7 (57.6)	0.001
Sedentary behaviors	242.1 (113.6)	342.6 (213.2)	267.5 (163.9)	221.2 (141.8)	466.6 (199.5)	200.8 (98.5)	0.001
Proportion of munites spent in physical activities related to							
Work	0.70 (0.35)	0.49 (0.42)	0.68 (0.37)	0.78 (0.34)	0.67 (0.39)	0.42 (0.35)	0.001
Transport	0.11 (0.22)	0.20 (0.32)	0.15 (0.24)	0.17 (0.30)	0.20 (0.28)	0.36 (0.30)	0.001
Recreation	0.19 (0.31)	0.31 (0.37)	0.17 (0.33)	0.05 (0.17)	0.13 (0.25)	0.23 (0.26)	0.001
Vigorous activities	0.42 (0.41)	0.23 (0.30)	0.53 (0.39)	0.51 (0.46)	0.29 (0.41)	0.18 (0.27)	0.001
Moderate activities	0.58 (0.41)	0.77 (0.30)	0.47 (0.39)	0.49 (0.46)	0.71 (0.41)	0.82 (0.27)	0.001
Percentage of participants doing no physical activities related to							
Work	43 (29.9%)	66 (42.0%)	36 (24.5%)	33 (19.0%)	46 (28.6%)	17 (23.9%)	0.001
Transport	92 (63.9%)	89 (56.7%)	69 (46.9%)	34 (19.5%)	67 (41.6%)	18 (25.4%)	0.001
Recreation	59 (41.0%)	65 (41.4%)	67 (45.6%)	156 (89.7%)	93 (57.8%)	23 (32.4%)	0.001
Vigorous activities	73 (50.7%)	88 (56.1%)	51 (34.7%)	83 (47.7%)	98 (60.9%)	41 (57.7%)	0.001
Moderate activities	34 (23.6%)	25 (15.9%)	27 (18.4%)	14 (8.0%)	25 (15.5%)	2 (2.8%)	0.001

a Categorical variables were analyzed by using Chi-squared test and expressed as numbers and percentages. Continuous variables were analyzed by using one-way ANOVA test and expressed as means and standard deviations.

b WHO recommendations on physical activity for health is at least 150 min per week of moderate-intensity physical activity or 75 min per week of vigorous-intensity physical activity or equivalent (at least 600 MET–minutes per week; MET, means the metabolic equivalent of task).

The proportion of weekly minutes spent in different physical activity domains (work, transport, and recreation) and vigorous-intensity and moderate-intensity physical activities from total weekly minutes spent doing physical activity is calculated. The means of the proportion of weekly minutes spent in physical activities related to work, transport, and recreation from total physical activity were 0.53 ± 0.42, 0.29 ± 0.36, and 0.19 ± 0.31, respectively. In the same way, the means of the proportion of weekly minutes spent in vigorous-intensity physical activities and moderate-intensity physical activities from total physical activity were 0.27 ± 0.38 and 0.73 ± 0.38, respectively. In addition, the percentages of participants who did not engage in any physical activity were recorded for various domains of physical activity. The percentages of participants who did not engage in any physical activity related to work, transport, recreation, vigorous-intensity and moderate-intensity physical activities were 39.9, 42.1, 58.2 62.7, and 16.9%, respectively.

Table 3 shows the risk of physical inactivity for all participants based on nationality, sociodemographic variables and BMI. Compared with participants from the Philippines, subject form several other countries had a significantly higher risk of being physically inactive, including Saudi Arabia [adjusted odds ratio (OR) = 3.01, *p* = 0.043], Yemen (adjusted OR = 3.26, *p* = 0.004), Syria (unadjusted OR = 2.94, *p* = 0.003), Jordan (adjusted OR = 2.24, *p* = 0.039), Turkey (adjusted OR = 5.56, *p* = 0.001), Pakistan (adjusted OR = 2.23, *p* = 0.004), Afghanistan (adjusted OR = 2.49, *p* = 0.027), India (adjusted OR = 5.28, *p* = 0.001), and Bangladesh (adjusted OR = 5.85, *p* = 0.001). Moreover, longer residency period in Saudi Arabia was significantly associated with a higher risk of physical inactivity (unadjusted OR = 1.03, *p* = 0.001). The participants those who live within a family household had a significantly higher risk of physical inactivity compared with those who live within non-family household (adjusted OR = 2.18, *p* = 0.001). Participants have at least a college degree had a significantly higher risk of physical inactivity compared with those with lower education level (adjusted OR = 1.45, *p* = 0.048). In the same fashion, participants having high monthly income (1,000 USD or more) had a significantly higher risk of physical inactivity compared with those who have low monthly income (unadjusted OR = 1.88, *p* = 0.001). Finally, Increasing BMI was significantly associated with a higher risk of physical inactivity (adjusted OR = 1.07, *p* = 0.001).

**TABLE 3:** Risk of physical inactivity among all participants (n = 1800) for sociodemographic characteristics and body mass index, Relationship between Obesity, Physical Activity, and Dietary Pattern among Men in Saudi Arabia Project, Saudi Arabia, 2019.

Variables	Unadjusted odds ratio^a^	95% CI	*p* value	Adjusted odds ratio^b^	95% CI	*p* value
Participants Nationality						
Filipino	1.00			1.00		
Saudi	7.46	3.65–15.25	**0.001**	3.01	1.04–8.72	**0.043**
Egyptian	1.25	0.59–2.66	0.564	0.84	0.38–1.85	0.666
Yemeni	4.50	2.15–9.44	**0.001**	3.26	1.45–7.36	**0.004**
Syrian	2.94	1.43–6.05	**0.003**	1.57	0.72–3.40	0.256
Jordanian	4.01	1.97–8.16	**0.001**	2.24	1.04–4.84	**0.039**
Sudanese	1.28	0.61–2.69	0.521	1.55	0.70–3.41	0.282
Turkish	3.84	1.92–7.66	**0.001**	5.56	2.53–12.23	**0.001**
Pakistani	3.08	1.49–6.38	**0.002**	2.23	1.44–7.21	**0.004**
Afghan	2.04	0.97–4.27	0.059	2.49	1.11–5.61	**0.027**
Indian	3.33	1.62–6.85	**0.001**	5.28	2.39–11.66	**0.001**
Bangladeshi	3.20	1.50–6.85	**0.003**	5.85	2.57–13.30	**0.001**
Age (years)	1.02	1.00–1.05	0.075	0.99	0.96–1.02	0.609
Residency period in Saudi Arabia (years)	1.03	1.02–1.04	**0.001**	1.00	0.98–1.03	0.709
Household Type						
Non-family household	1.00			1.00		
Family household	1.16	0.90–1.50	0.246	2.18	1.48–3.22	**0.001**
Marital Status						
Single	1.00			1.00		
Married	1.01	0.75–1.36	0.940	0.77	0.54–1.10	0.155
Education Level						
High school or less	1.00			1.00		
College degree or more	1.45	1.19–1.78	**0.001**	1.45	1.00–2.08	**0.048**
Monthly Income						
Low (<1000 USD)	1.00			1.00		
High (≥1000 USD)	1.88	1.55–2.29	**0.001**	1.07	0.81–1.43	0.631
Body Mass Index (kg/m^2^)	1.06	1.03–1.09	**0.001**	1.07	1.04–1.10	**0.001**

a Univariate logistic regression analysis was used to test differences between physically inactive participants versus physically active participants (reference group). Differences were considered statistically significant at *p* value <0.05 and significant values were presented in **Bold type**.

b Multivariate logistic regression analysis was used to test differences between physically inactive participants versus physically active participants (reference group) after adjusting for participants’ sociodemographic characteristics and body mass index. Differences were considered statistically significant at *p* value <0.05 and significant values were presented in **Bold type**.

## Discussion

This study investigated the physical activity levels of a multi-ethnic sample of middle-aged men living in Saudi Arabia. About one-third of the participants are physically inactive. On a worldwide scale, Saudi Arabia has a high prevalence of physical inactivity [24–26]. A population-based national survey revealed that 66.6% of the population in Saudi Arabia (60.1% of males and 72.9% of females) were physically inactive [24]. Another study reported that 96.1% of Saudi adults aged 30–70 years were physically inactive [25]. A recent population-based study reported that the prevalence of physical inactivity was 82.6% among Saudi citizens (71.7% of males and 91.1% of females) and 86.1% among non-Saudi residents (83.9% of males and 92.0% of females) aged 15 years or more [26]. Several barriers, such as the absence of motivation, growing urbanization, crowded traffic, hot desert weather, cultural obstacles, absence of social support, and inadequate time and resources, prevent regular physical activity among people in Saudi Arabia [9].

The current study found considerable differences in physical activity levels among participants from different countries. Several lifestyle factors, such as work type, transportation, leisure time activities, and the intensity and duration of physical activity, could be accountable for these differences [11]. Manual labor jobs such as farming, housekeeping, and culinary are often associated with higher physical activity levels than office labor jobs such as the secretary, data entry and accounting [27]. For example, the majority of middle-aged Saudi men work in office labor occupations. However, the majority of middle-aged Afghan men living in Saudi Arabia work in manual labor occupations. Moreover, typical modes of transportation can have an impact on people’s levels of physical activity. Walking or riding a bicycle has been linked to higher levels of physical activity when compared to using automobiles for short-distance commuting [28]. For example, the vast majority of middle-aged Bangladeshi men living in Saudi Arabia use bikes frequently to commute short travels. In contrast, middle-aged Saudi men depend mainly on cars for transportation, even for short-distance trips. The leisure-time physical activities of middle-aged men from diverse nations are influenced by cultural standards, available free time, and resources and appropriate sites availability for completing workouts and engaging in recreational physical activities [29]. Our results showed that middle-aged Saudi men, for example, were found to be more involved in recreational physical activities than middle-aged Sudanese men. Fortunately, examining these differences in physical activity characteristics can help identify and implement relevant approaches to reduce physical inactivity in high-prevalence groups.

Monitoring factors associated with physical inactivity for various population subgroups is an essential part of health-promoting activities to reduce physical inactivity [30]. According to our findings, several sociodemographic characteristics were shown to be connected with increased physical inactivity risk. One of these characteristics was nationality, which might be attributed to cross-cultural differences in jobs, modes of transportation, and lifestyles, including typical leisure activities, among participants from different countries [14]. Longer residency in Saudi Arabia was linked to a higher risk of physical inactivity, which could be explained by the country’s urbanization and motorization and their impact on people’s lifestyles [11]. This outcome was consistent with findings from prior studies from Saudi Arabia [18, 31]. Emigrants’ health is assumed to deteriorate with the length of time they spend in a new host country due to cultural variances, social and financial changes, as well as lifestyle modifications related to usual diet and physical activity level [32]. Our results showed that physical inactivity was found to be linked with living in a family household. In Saudi Arabia, social gatherings are a typical element of family life. Unfortunately, these get-togethers tend to center on sedentary activities like sharing meals and watching television [9]. Moreover, physical inactivity was found to be linked with higher education and income. In Saudi Arabia, educated and/or financially stable men generally work in office occupations that require them to sit for long periods of time and rely on cars for mobility, which can lead to physical inactivity [27].

Saudi Arabia has one of the highest overweight and obesity rates worldwide [10]. Obesity is associated with the incidence of several chronic diseases and health disorders [33, 34]. It is recognized that physical inactivity is a significant factor that contributes to overweight and obesity incidence [35]. The Saudi population’s high rates of overweight and obesity can be attributed to a high prevalence of physical inactivity and sedentary lifestyles [36]. According to our findings, a greater BMI was associated with a higher risk of physical inactivity among the participants. This outcome was in line with prior research findings reported in Saudi Arabia [25, 27, 37–39].

Several projects were launched in Saudi Arabia to promote physical activity. The majority of them were disconnected, short-term initiatives that lacked a coordinating body and objective evaluations of their results. A national policy that fosters active living while discouraging sedentary behavior is required, including input from all stakeholders [40]. The healthy city initiative is one of these projects applied in twenty-five Saudi cities and involved various activities, including awareness about the health benefits of physical activity, and friendly roads and public places for walking for people of all ages [40]. The ministry of health in Saudi Arabia established many programs for physical activity promotion for various population subgroups around the country. These programs included a range of activities, including lectures, workshops, training courses for health care professionals, brochures, posters, and media awareness campaigns [40]. The Saudi national transformation program of the vision 2030 is a strategic plan established in Saudi Arabia to develop various public service sectors, including public health. This program is targeting healthy lifestyle promotion and public participation in physical activity at the population level [40].

The WHO Global Action Plan on Physical Activity 2018–2030 was developed to ensure that everyone has access to safe and enabling environments, as well as a variety of opportunities to be physically active in their daily lives, as a means of improving individual and community health and contributing to all nations’ social, cultural, and economic development [5]. This action plan comprises a combination of guiding principles, strategic objectives and recommended policy actions designed to disseminate physical activity culture at the population level [5]. Each country is encouraged to determine a strategic combination of these policy actions for execution over the short, medium, and long terms to promote physical activity in different members of the community [5]. The chosen policy actions should be adjusted to the demands of varying population subgroups based on the country context. Consequently, each country needs to evaluate the physical activity levels of various population subgroups to examine gaps and relevant policy actions that may be enhanced the current situation [5]. This study’s outcomes provide for decision-makers in Saudi Arabia an evaluation of physical activity levels for a multi-ethnic population of middle-aged men and factors associated with physical inactivity.

There are some limitations to this study that should be taken into account. A cross-sectional design has the disadvantage of being unable to discern causality. The second limitation is that the main variables, physical activity levels, are self-reported outcome measures susceptible to recall and social desirability biases. Finally, because our data is limited to Riyadh, we may not generalize our findings to the rest of Saudi Arabia. Nevertheless, the current study still provides relevant information about physical activity levels and factors associated with physical inactivity in a multi-ethnic sample of middle-aged men living in Saudi Arabia.

In conclusion, physical inactivity was found to be considerably prevalent among middle-aged men in Saudi Arabia. Findings revealed significant differences in physical activity levels among middle-aged men from twelve Middle Eastern and Asian countries living in Saudi Arabia. Physical inactivity risk is substantially linked with nationality, residency length in Saudi Arabia, household type, education level, monthly income, and BMI among a multi-ethnic sample of middle-aged men in Saudi Arabia.
